# Disseminated coccidioidomycosis in a patient with juvenile idiopathic arthritis receiving infliximab

**DOI:** 10.1186/s12969-021-00549-7

**Published:** 2021-05-01

**Authors:** Megan Trainor, Emily Henkel, Lucia Z. Diaz, Ruy Carrasco

**Affiliations:** 1 Division of Dermatology, Dell Medical School, University of Texas at Austin, 601 E 15th St, CEC C2.470 , Austin, TX 78701 USA; 2 Long School of Medicine, University of Texas Health Science Center at San Antonio, Texas San Antonio, USA; 3 Dell Children’s Medical Center, Texas Austin, USA; 4 Department of Pediatrics, Dell Medical School, University of Texas at Austin, Austin, Texas USA; 5 Presbyterian Healthcare Services, New Mexico Albuquerque, USA

**Keywords:** *Coccidioides immitis*, Corticosteroids, Disseminated coccidioidomycosis, Coccidioidomycosis, Juvenile idiopathic arthritis, Infliximab, Methotrexate, Tumor necrosis factor alpha (TNF-α) inhibitors

## Abstract

**Background:**

*Coccidioides immitis* is a dimorphic fungus endemic to the arid climates of the Southwest United States, Mexico and parts of Central and South America. Human infection occurs through inhalation of spores with less than half of exposures progressing to a symptomatic state that primarily consists of pulmonary manifestations. Disseminated coccidioidomycosis is exceedingly rare, occurring in fewer than 1 % of symptomatic infections. Through hematogenous spread, the fungus can infect most organ systems and may be fatal without systemic antifungal treatment. Individuals with impaired cell-mediated immunity either from primary immunodeficiency disorders or secondary to immunosuppression with medications such as tumor necrosis factor alpha (TNF-α) inhibitors have increased risk of disseminated coccidioidomycosis and previous cases of coccidioidomycosis have been reported with biologic therapy.

**Case presentation:**

We present a case of disseminated coccidioidomycosis in a 16-year-old female with polyarticular juvenile idiopathic arthritis (JIA) being treated with prednisone, methotrexate, and infliximab. The patient presented with symptoms of meningeal irritation, bilateral choroidal lesions, and necrotizing peripheral pneumonia. Her infection was thought to be a reactivation of coccidioidomycosis given her history of resolved pneumonia that occurred after traveling to Arizona, New Mexico, and El Paso one year prior to presentation. Following diagnosis, she improved with discontinuation of her immunosuppressive medications and two weeks of intravenous amphotericin B and fluconazole with plans for lifetime treatment with fluconazole while immunosuppressed. Due to worsening arthritis, she will begin tofacitinib and continue close monitoring of chest x-rays and coccidioides antibody.

**Conclusions:**

Patients undergoing immunosuppressive therapy for rheumatological conditions are at increased risk of disseminated coccidioidomycosis and should be evaluated with high suspicion when presenting with atypical symptoms and history of travel to endemic regions.

## Background

Coccidioidomycosis is a spectrum of disease caused by the inhalation of *Coccidioides immitis* arthroconidia in the endemic regions of the Southwest United States, Mexico, and parts of Central and South America [1]. It is estimated that there are around 150,000 cases of *Coccidioides* infection in the United States annually with 95 % self-resolving and fewer than 1 % progressing to disseminated disease [2, 3]. Common symptoms of primary infection include a flu-like illness with cough, fever, pleuritic chest pain, and rash [4]. Given the pulmonary manifestations of primary infection with *Coccidioides*, studies suggest the true prevalence of coccidioidomycosis is underestimated and as much as 30 % of diagnosed community-acquired pneumonias (CAP) in southern Arizona are caused by coccidioidomycosis [3].

Extra-pulmonary disseminated disease occurs through hematogenous spread that involves the skin, bones, central nervous system, and many other organ systems [4, 5]. Infection of the central nervous system places the patient at highest risk of mortality and must be promptly identified and treated [4]. Risk factors for disseminated coccidioidomycosis include human immunodeficiency virus (HIV), transplant, diabetes, pregnancy, and immunosuppressive medications [1].

Diagnosis of coccidioidomycosis can be made using serologic tests such as enzyme immunoassay, latex particle agglutination, and immunodiffusion [4, 6]. Titers of IgG complement-fixing antibodies are also useful in monitoring treatment response [6]. Additionally, the organism may be isolated on culture media or directly examined from sputum or bronchoalveolar lavage samples [4].

Fungal infections with *Aspergillus*, *Coccidioides*, *Histoplasma*, *Cryptococcus*, *Sporothrix*, and *Candida* have been reported during treatment with biologic agents [7]. Histoplasma is the most common invasive fungal organism identified and presents with nonspecific signs and symptoms such as cough, fever, chills, weight loss and possible rash [7].

Here we report the case of a 16-year-old female with juvenile idiopathic arthritis on immunosuppressive medications, including infliximab and methotrexate, who was found to have disseminated coccidioidomycosis.

## Case presentation

**Fig. 1:**
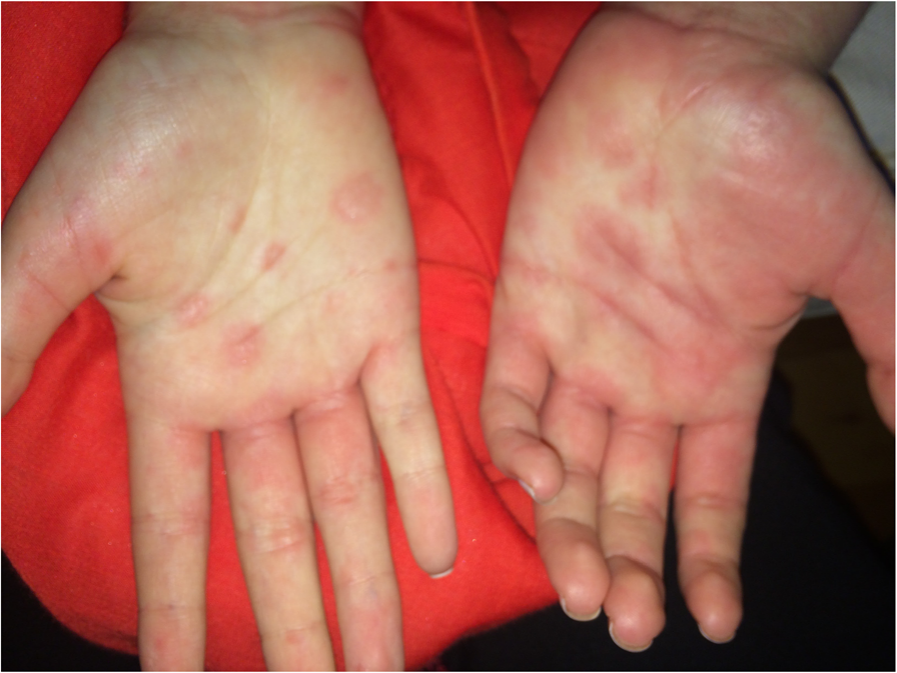
Urticarial pink plaques on bilateral hands

**Fig. 2:**
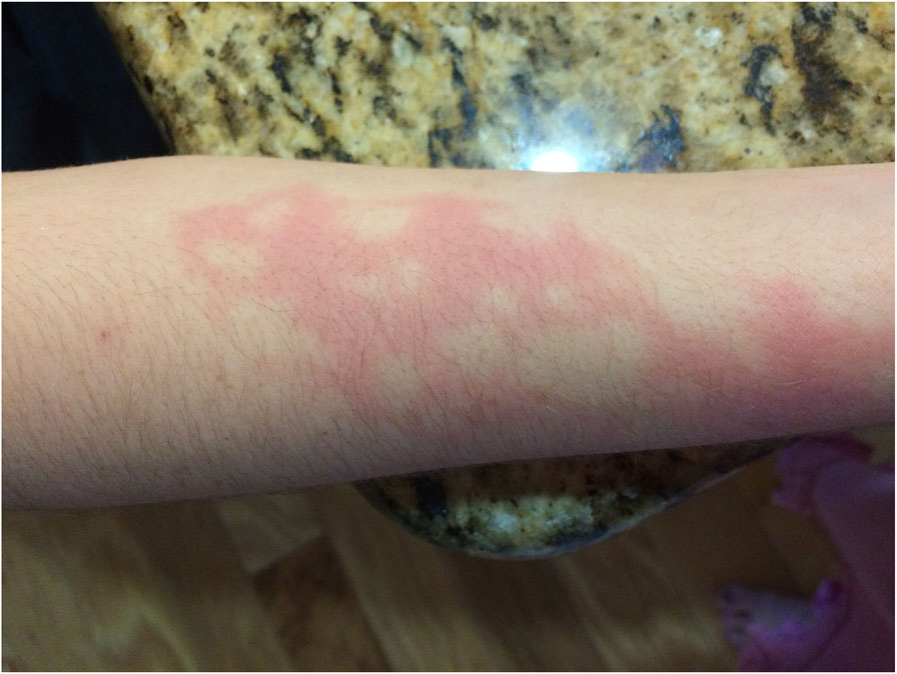
Urticarial pink plaques on right forearm

 A 16-year-old female with a history of hypothyroidism, fibromyalgia and polyarticular juvenile idiopathic arthritis presented to the emergency department with a three-week history of frontal headaches worsening in the preceding week accompanied by photophobia, phonophobia, emesis and nighttime fevers to 38.9^o^C. Additionally, she reported one month of ocular pain with a “black spot” in her left field of vision that began shortly after attending summer camp at a farm in Alabama. One week prior to presentation, she was evaluated by ophthalmology and found to have facial numbness and weakness and diagnosed with chorioretinitis. The initial concern by her retinal specialist was acute multifocal placoid pigment epitheliopathy (AMPEE), an autoimmune chorioretinal disease which can be complicated by neurologic involvement, including headaches. Several months prior to presentation the patient has transient urticarial eruptions on the extremities and trunk that were asymptomatic (Figs. 1 and 2). Further history revealed that seven months prior to presentation she had contracted pneumonia after traveling to Arizona, New Mexico, and El Paso. The pneumonia resolved with antibiotic treatment.

She had a four-year history of polyarticular juvenile idiopathic arthritis and her anti-nuclear antibody (ANA) titer ranged from 1:320 to 1:640 in a homogenous or speckled pattern. At the time of presentation, her arthritis was being treated with 10 mg of prednisone daily, 10 mg of methotrexate weekly and 10 mg/kg infliximab infusions every six weeks. She was initially treated with 10 mg/kg abatacept infusions every four weeks, but she was switched to infliximab after two years of therapy as she stopped responding to abatacept. Infliximab was selected as the patient declined injectable medication and preferred intravenous infusions. She was also undergoing occupational therapy.

**Fig. 3:**
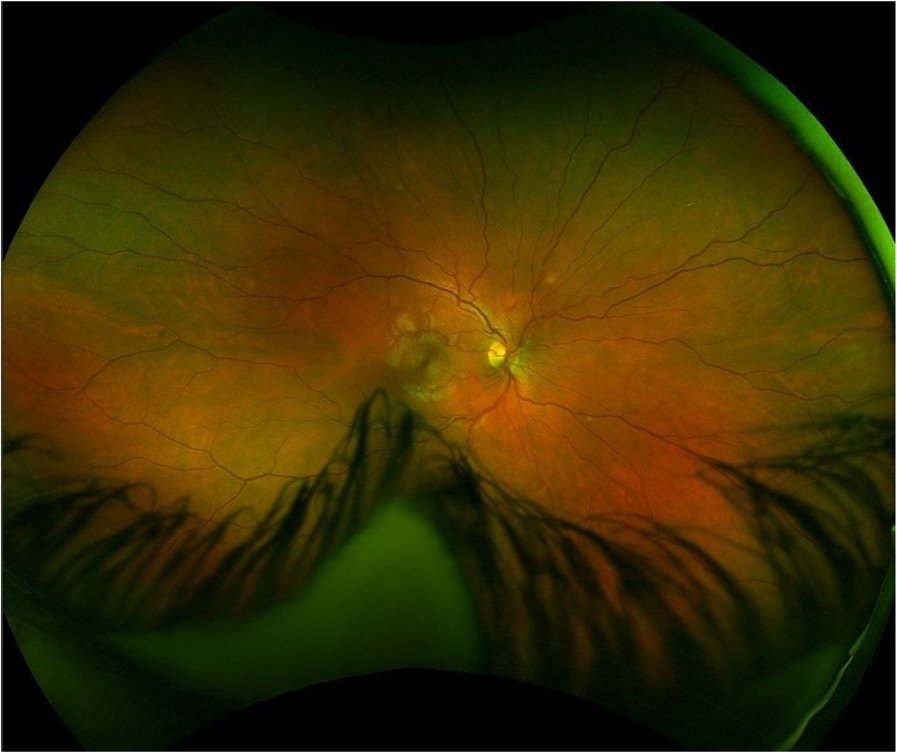
Fundoscopic exam with focal choroidal lesions

**Fig. 4:**
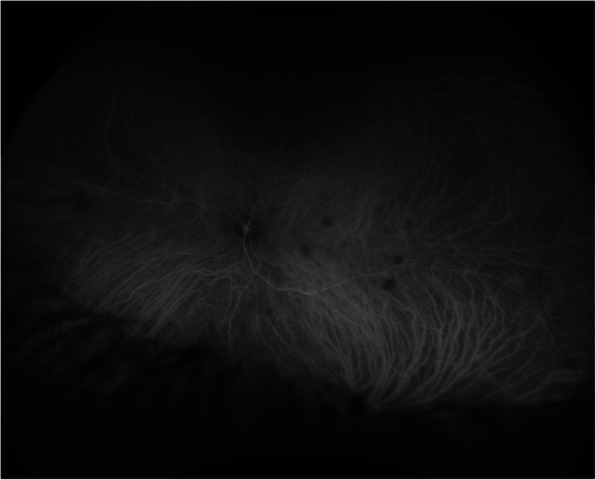
Indocyanine angiography highlights focal choroidal lesions

**Fig. 5:**
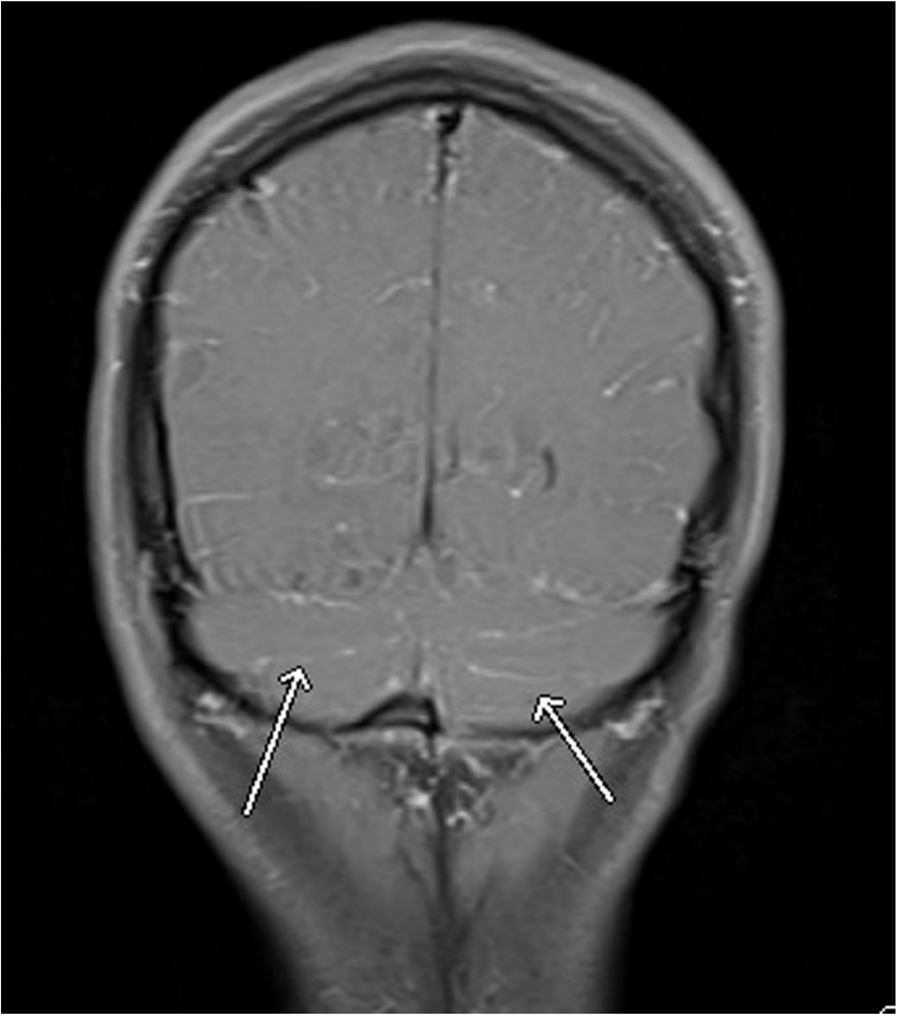
Brain FLAIR MRI with leptomeningeal enhancement

**Fig. 6:**
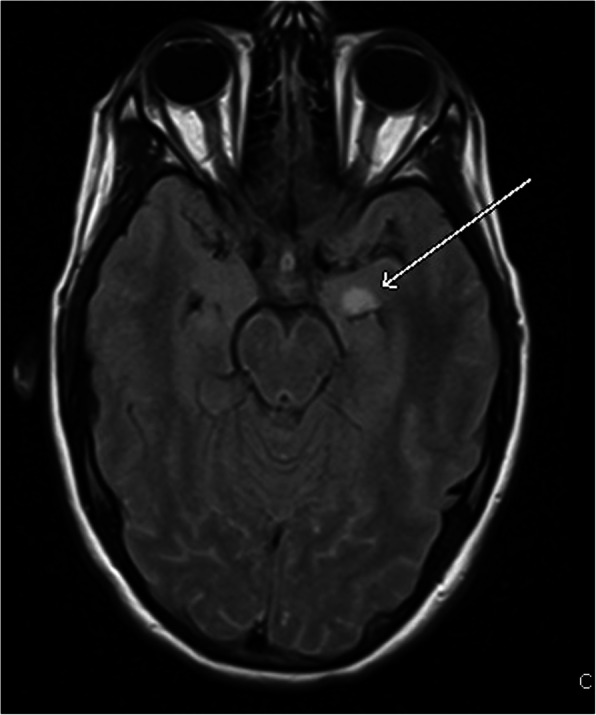
Brain FLAIR MRI with medial left temporal lobe enhancement

**Fig. 7:**
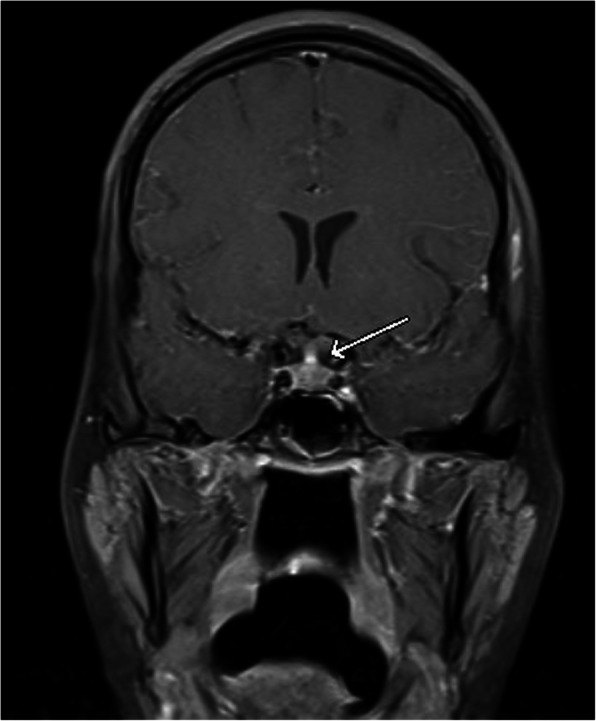
Brain T1-weighted MRI with a thickened and infiltrated infundibulum

**Fig. 8:**
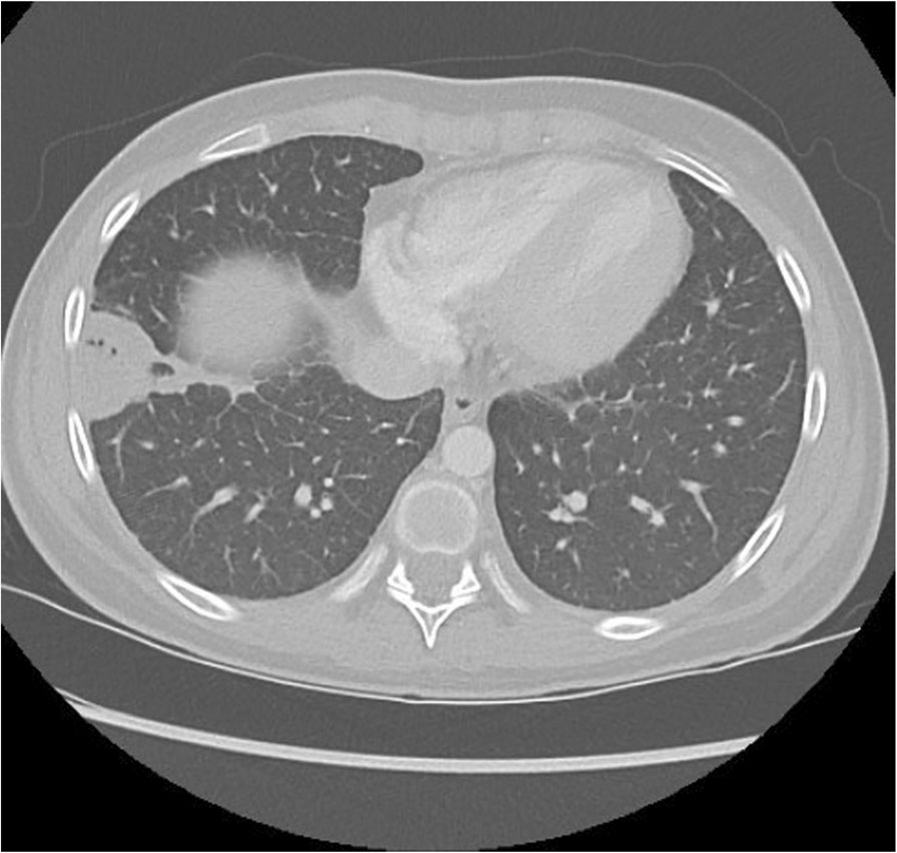
Chest CT with 3 × 4 cm right basilar lung consolidation with areas of developing necrosis

Slit lamp and optical coherence tomography (OCT) exams upon presentation revealed nonspecific bilateral choroidal lesions (Figs. 3 and 4). Brain and spine MRI showed enhancement of the left temporal lobe, a small infarct in the left medial temporal area, and a thickened and infiltrated infundibulum (Figs. 5, 6 and 7). Although the patient complained of headaches, the history was complicated by her worsening fibromyalgia. These MRI findings and multiple ophthalmic exams initially resulted in a diagnosis of AMPEE by retinal specialist. Additional imaging revealed a right lower lobe necrotizing pneumonia (Fig. 8). Her white blood cell (WBC) count was 11.7 and her erythrocyte sedimentation rate (ESR) was 46. Her cerebrospinal fluid (CSF) culture, right lung aspirate culture, and urine culture were all positive for *Coccidioides immitis*. Serology revealed *Coccidioides* IgG of 1:256 and positive *Coccidioides* IgM. She had positive CSF serology for cocci with complement-fixation antibody 1:4, IgG 7.6, IgM 3.0. Her right lung biopsy demonstrated necrotizing granulomatous inflammation with fungal organisms compatible with *Coccidioides*. Her serology was negative for Bartonella, Toxoplasma, cytomegalovirus, herpes simplex virus. Her T-spot was also negative. She was diagnosed with disseminated coccidioidomycosis with *Coccidioides* meningitis.

The patient was treated with IV fluconazole and amphotericin B for two weeks and has continued treatment with oral fluconazole, initially on 800 mg daily and tapered to 400 mg daily based with plans for her to continue with lifetime fluconazole based on the 2016 Infectious Diseases Society of America (ISDA) Clinical Practice Guideline for the Treatment of Coccidioidomycosis [8]. We. She stopped medications for her JIA during the admission to evaluate a disseminated infection v autoimmune condition such as AMPEE. She was also weaned off prednisone. Recent imaging demonstrated worsening of her arthritis off therapy, and she was started on hydroxychloroquine 200 mg twice daily with close clinical and lab monitoring. The patient was evaluated by a local infectious disease physician and an infectious disease physician at the National Institutes of Health who both recommended against the use of biologic medication given the systemic involvement of *Coccidioides*. She continues to have chest x-rays and brain MRIs every six months and *Coccidioides* antibody by complement fixation and immunodiffusion every three months. Her most recent lumbar puncture had normal white count, glucose, protein, and negative *Coccidioides* by immunodiffusion any by complement fixation. Her serology *Coccidioides* IgG has improved from 1:256 to 1:2. She currently has no neurologic sequalae and her vision has returned to baseline. She continues to follow with pediatric infectious disease, rheumatology, and ophthalmology.

## Discussion

Preserved cell-mediated immunity is critical to successful response to infection with *Coccidioides* [6]. TNF, an essential component to the immune system’s response, is released by macrophages and T cells to stimulate the release of cytokines that promote the creation and maintenance of granulomas [9]. The disruption of this process by TNF-α inhibitors is well-documented and data suggests patients on these medications are at increased risk for infection and reactivation of granulomatous diseases [9]. Studies suggest that infliximab is associated with greater risk of granulomatous disease reactivation than commonly used etanercept [9, 10].

One case series performed in a *Coccidioides* endemic region demonstrated that 12 of 13 adult cases of coccidioidomycosis were patients receiving infliximab treatment, 11 of whom were also taking methotrexate [10]. Infliximab was shown to have a statistically significant increase in symptomatic coccidioidomycosis, even following adjustment for methotrexate cotreatment [10]. Additionally, a retrospective study found an increase in the risk of symptomatic coccidioidomycosis in patients treated with infliximab, with two disseminated coccidioidomycosis cases occurring in adult patients treated with prednisone monotherapy [11].

To our knowledge, this is the first report of disseminated coccidioidomycosis in a patient with juvenile idiopathic arthritis on immunosuppressive therapy. Our patient was on triple immunosuppressive therapy of infliximab, prednisone, and methotrexate at the time of presentation, putting her at increased risk of disseminated disease. Given our patient’s history of pneumonia following vacationing to an endemic region it is possible she was infected one year prior and either slowly progressed to a disseminated infection or underwent reactivation upon reaching an immunosuppressive threshold. Similar cases of disseminated coccidioidomycosis have been documented presenting months to years following travel to endemic regions [12, 13]. This emphasizes the need for in depth patient interviewing and eliciting a comprehensive travel history in immunosuppressed patients presenting in non-endemic regions. This case also demonstrated ocular involvement from dissemination, which is a unique presentation [12, 14].

In summary, TNF-α inhibitors and other immunosuppressive agents are widely utilized for a variety of rheumatological conditions. With the advancement of therapeutic immunosuppression, more patients are becoming at risk for rare opportunistic infections. When evaluating atypical symptomatic patients on immunosuppressive therapy healthcare providers must elicit thorough travel histories and maintain wide differentials to ensure prompt treatment of potentially fatal infections.

## Conclusions

We describe a case of disseminated coccidioidomycosis in a 16-year-old female taking immunosuppressive agents for polyarticular juvenile idiopathic arthritis who traveled to an endemic region one year prior to presentation. Our findings demonstrate that immunosuppressive treatment, especially infliximab, in patients with rheumatic conditions are at a higher risk of reactivation and disseminated coccidioidomycosis. This case highlights the need for obtaining thorough travel history and maintaining high suspicion when evaluating an immunosuppressed patient presenting with atypical symptoms.

## Data Availability

Data sharing not applicable to this article as no datasets were generated or analyzed during the current study.

## Abbreviations

JIA: Juvenile idiopathic arthritis
TNF-α: Tumor necrosis factor alpha
CAP: Community-acquired pneumonia
AMPEE: Acute multifocal placoid pigment epitheliopathy
ANA: Anti-nuclear antibody
OCT: Optical coherence tomography
WBC: White blood cell
ESR: Erythrocyte sedimentation rate
CSF: Cerebrospinal fluid
